# Two alleles of *unc-52* locus disrupting potential cell-binding motif of UNC-52

**DOI:** 10.17912/micropub.biology.000250

**Published:** 2020-05-17

**Authors:** Rachel Wilsey, Sabrina Hodge, Krysta Kenney, Jacob Wahl, Roshni Jaffery, Avery Brau, Zhongqiang Qiu, Myeongwoo Lee

**Affiliations:** 1 One Bear Place 97388, Department of Biology, Baylor University, Waco, TX 76798, U.S.A

**Figure 1:**
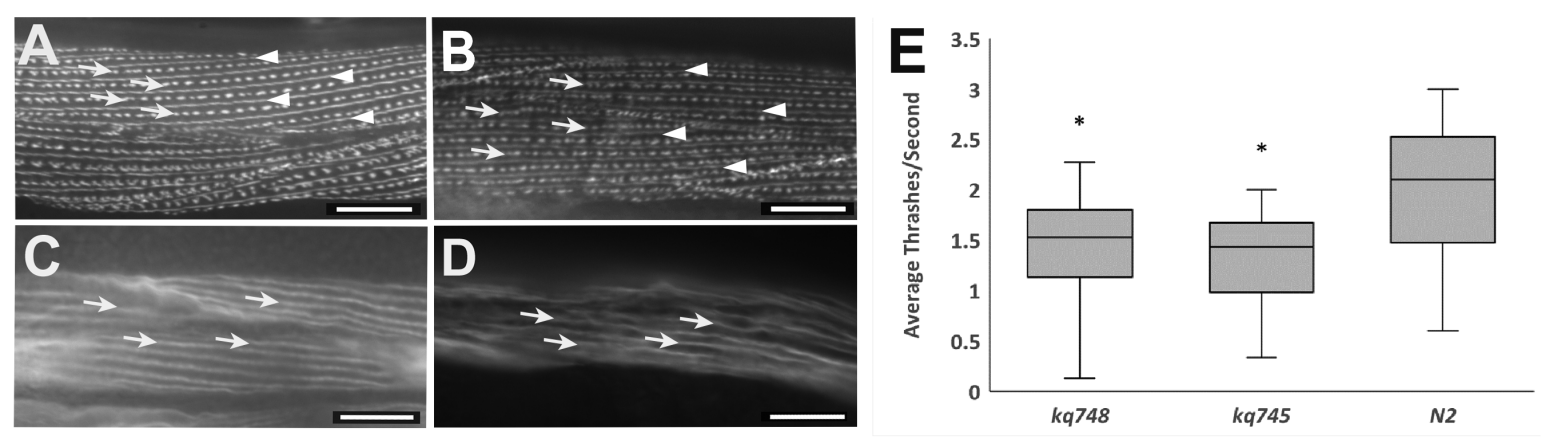
**A:** Muscle cell of NK358 *pat-3::GFP* animal.The dotted lines represent dense bodies (arrows) and straight lines represent M-lines (arrowheads); **B:** Muscle cell of a *pat-3::GFP; unc-52(kq748)* animal. The dotted lines represent dense bodies (arrows) and straight lines (arrowheads) represent M-lines. Localization appears similar to Figure 1A; **C:** Rhodamine-conjugated phalloidin staining of an N2 muscle cell. Actin cytoskeletons along the length of muscle are stained (arrows); **D:** Rhodamine-conjugated phalloidin staining of an *unc-52(kq748)* muscle cell. No obvious abnormalities in thin (actin) filaments (arrows) are present. Scale bar = 10 µm.; **E:** Thrashing assay results for *unc-52 (kq748)* (1.4454 average thrashes/second, n=50), *unc-52(kq745)* (1.339 average thrashes/second, n=50), and N2 wild-type (1.99 average thrashes/second, n=50). * p-value < 0.05 compared to N2 wild-type.

## Description

The *unc-52* gene in *Caenorhabditis elegans* encodes for the protein UNC-52 and is a homolog of the mammalian gene Heparan Sulfate Proteoglycan 2 (HSPG2), which encodes for the protein perlecan (Rogalski **et al.*,* 1993; Mullen **et al.*,* 1999). HSPG2 is implicated in the human diseases Schwartz-Jampel Syndrome type 1 and Dyssegmental Dysplasia, Silverman-Handmaker type (Arikawa-Hirasawa **et al.*,* 2001; Stum **et al.*,* 2006). The mutated *unc-52* gene expresses the phenotype for uncoordinated movement (“Unc”), which involves progressive paralysis and retarded sarcomere construction (Martinez **et al.*,* 2018). The UNC-52 protein is localized in the striated muscle dense bodies and the basement membrane, where it plays an important role in developmental processes such as cell adhesion, cell migration, and signal transduction (Kihira **et al.*,* 2012). Within the *unc-52* gene, an RGD (Arg- Gly- Asp) sequence is located at amino acid locations 746, 747, and 748 in exon 7. Exon 7, containing RGD^748^, is included in all three major isoforms, short (S), medium (M), and long (L) (Mullen *et al.*, 1999). The RGD sequence is part of the Laminin IV type A domain and primarily functions as a cell attachment and adhesion site for integrins (Rogalski **et al.*,* 1993; Mullen **et al.*,* 1999). In this study, two separate mutations were performed on this *unc-52* RGD sequence using CRISPR-Cas9 technology. The *unc-52(kq748)* mutation replaced the aspartic acid (D) located at the 748 amino acid position with a glutamic acid (E) (Takahashi **et al.*,* 2007). The *unc-52(kq745)* mutation removed the RGD sequence by deleting amino acids 746, 747, and 748. Previous studies have shown that *unc-52* gene mutations cause the disorganized distribution of *pat-3* β integrin, which is a receptor for extracellular matrix proteins (Rogalski **et al.*,* 1995). In order to study the cellular phenotypes of the *unc-52(kq748)* mutation, a double mutant was created: *pat-3::GFP; unc-52 (kq748)*. Staining showed that localization of the pat-3::GFP reporter in the double RGD mutant appeared normal, with dense bodies and M-lines alternating along the muscle filaments (Figures 1A and 1B). In order to visualize the actin filaments in the body wall muscles of N2 wild type and *unc-52(kq748)* mutants, staining was performed using 0.4 U/mL rhodamine-conjugated phalloidin, Thermo Fisher Scientific, Waltham, MA (Figures 1C and 1D). This *unc-52(kq748)* staining showed no obvious abnormalities outside of interruptions due to fixation and slicing procedures (Figure 1D). Additional assays were performed to investigate any other physical or behavioral phenotypes. The thrashing assay, in which worms were placed in drops of M9 buffer and the number of thrashes were counted for 15 seconds, showed a 27% average decrease in thrashes per second for the *unc-52(kq748)* mutants compared to the N2 worms, and a 33% decrease in *unc-52(kq745)* mutants (Figure 1E). A one-way ANOVA statistical test was performed to check the statistical significance of these results. The p-value from the ANOVA test was 3.1×10^-9^, which is less than the 0.05 needed to be statistically significant. Post hoc testing was then done to see which groups differed. Both the *unc-52(kq748)* and *unc-52(kq745)* groups differed significantly from the N2 wild-type group, as shown by p-values of 7.1×10^-6^ and 4.63×10^-8^ respectively. These results show that there was a statistically significant decrease in motility for both *unc-52(kq748)* and *unc-52(kq745)* mutants.

## Methods

CRISPR-Cas9 technology was used to create both the *unc-52(kq748)* and *unc-52(kq745)* mutations. For the *unc-52(kq748)* mutation, the DNA repair template (UNC52RGE748), crRNA (UNC52RGE748), tracrRNA (cat. #1072532), Cas9 nuclease (cat. #1081058), and a *dpy-10* crRNA co-CRISPR marker were mixed and micro-injected into wild-type N2 worms, which served as the parent (P0) generation. F1 worms that carried the mutation were then identified and isolated using the *dpy-10* marker (Arribere **et al.*,* 2014; Paix **et al.*,* 2015) and underwent PCR genotyping using mutant specific primers (UNC52RGE748SEQF and UNC52RGE748SEQR) to screen for the RGE mutation. The F2 generation then also underwent PCR screening using both a mutant specific primer (UNC52RGE748SEQF) and a wild-type specific primer (UNC52RGD748WTF) to isolate non-dumpy homozygous mutants. These homozygous mutant PCR products were sequenced to validate the success of the mutation (Psomagen Inc, Rockville, MD). This process was repeated for the *unc-52(kq745)* mutation using the DNA repair template (UNC52RGD748D) and the primers (UNC52RGD748DF and UNC52RGDWTF). The DNA repair template and all other oligos were designed at IDT Inc., Coralville, IA. A double mutant was then created by crossing *unc-52(kq748)* II males with NK358 *pat-3::GFP* III hermaphrodites. Homozygous double mutants from the F2 generation were identified as green-fluorescent progeny and confirmed through PCR genotyping. Staining was performed by collecting mutants and fixing them with ice-cold methanol for 5 minutes. Images were captured using a Nikon Eclipse Ni-U epifluorescence microscope and processed with NIS Elements (version 5.02). Once the homozygous mutants were identified and isolated, phenotypic assays were conducted on 50 young adult worms from both homozygous mutants and N2 wild-type worms in order to characterize the phenotypes for both the *unc-52(kq748)* and *unc-52(kq745)* mutations. A one-way ANOVA test was then run to confirm statistical significance of the results. Since the p-value for the ANOVA test was less than 0.05, post hoc tests were run to determine which groups differed (JMP, version 15, Cary, NC).

crRNA sequences (5’ to 3’)

UNC52RGD748 TTTCTATTGATTATGCTCGT

(dpy-10) ZQDP10A GCUACCAUAGGCACCACGAG

Repair templates (5’ to 3’)

**Table:** 

UNC52RGE748	caaaaatatgtgttcaaccatcactttcttcagGTGTCAATCGACTACGCAAGAGGCGAACGTGACCAGCTCGAGCTCACCACCTCGGACTCCCG
UNC52RGD748D	caaaaatatgtgttcaaccatcactttcttcagGTGTCAATCGACTACGCAAGAGATCAACTCGAGCTCACCACCTCGGACTCCCGCCAACCATA

Primers

UNC52RGE748SEQF TTCCTTGCTTCTGCTCAGGT

UNC52RGE748SEQR TGATCGGAGTTGCCATTTCCA

UNC52RGD748WTF TTCTATTGATTATGCTCGTGGGGAT

UNC52RGE748F TCAATCGACTACGCAAGAGGC

UNC52RGD748DF GTCAATCGACTACGCAAGAGATCAA

## Reagents

BU748 *unc-52(kq748)* II, BU745 *unc-52(kq745)* II, and BU111 *unc-52(kq748); pat-3::GFP* are available upon request. NK358 *pat-3::GFP* III was purchased from Caenorhabditis Genetics Center, Minneapolis, MN.
